# Effect of Boiling and roasting on lipid quality, proximate composition, and mineral content of walnut seeds *(Tetracarpidium conophorum)* produced and commercialized in Kumba, South‐West Region Cameroon

**DOI:** 10.1002/fsn3.570

**Published:** 2017-12-20

**Authors:** Fabrice Tonfack Djikeng, Edem Selle, Azia Theresia Morfor, Bernard Tiencheu, Blaise Arnaud Hako Touko, Gires Teboukeu Boungo, Serges Ndomou Houketchang, Mallampalli Sri Lakshmi Karuna, Michel Linder, François Zambou Ngoufack, Hilaire Macaire Womeni

**Affiliations:** ^1^ School of Agriculture and Natural Resources Catholic University Institute of Buea Buea Cameroon; ^2^ Department of Biochemistry Faculty of Science University of Dschang Dschang Cameroon; ^3^ Department of Biochemistry Faculty of Science University of Buea Buea Cameroon; ^4^ Centre for Lipid Research CSIR‐Indian Institute of Chemical Technology Tarnaka Hyderabad India; ^5^ Biomolecular Engineering Laboratory (LIBio) University of Lorraine ENSAIA Vandoeuvre‐les‐Nancy France

**Keywords:** African walnut, boiling, oil quality, proximate composition, roasting

## Abstract

The effect of boiling and roasting on the lipid quality, proximate composition, and mineral content of African walnut seeds (*Tetracarpidium conophorum*) was assessed. Results indicated that the quality of walnut oil significantly (*p* < .05) reduces with the treatments. Oils extracted from *DBWN* 60* *min (Dried and boiled walnuts 60 min) and *FBWN* 60 min (Boiled fresh walnuts 60 min) were the most altered. The proximate composition and mineral content of walnut seeds was also significantly affected (*p* < .05) by the treatments. This study reveals that, thermal processing has significant effects on the nutrients and quality of lipids of walnut oil. *DTRWN* 60 min (Dried and traditionally roasted walnuts 60 min), *DORWN* 60 min (Dried and oven roasted walnuts 60 min), and *TRFWN* 30 min (traditionally roasted fresh nuts 30 min) are the best methods for cooking walnut because they preserve the quality of its lipids and some of the nutrients.

## 1 INTRODUCTION

African walnut (*Tetracarpidium conophorum*) is a popular Central and Western Africa plant where it is planted mainly for its nuts, which are usually eaten as snacks when the unshelled nuts are boiled and cracked (Babalola, 2011). The proximate composition of *T. conophorum* revealed that it is rich in protein (29.14%), fat (54.14%), carbohydrate (4.17%), ash (3.32%), and several vitamins (Arinola & Adesina, 2014). A bitter taste is usually observed upon drinking water immediately after eating the nuts, due to the presence of antinutrients. The leaves, barks, and nuts of *T. conophorum* have also been demonstrated as good sources of phenolic antioxidants with various biological properties. Food materials are usually processed in order to improve palatability and reduce toxicity, and as a means of preservation (Kanu, Kalu, & Okorie, 2015). Walnuts seeds are generally consumed after several processing techniques. General method of processing of walnut prior to consumption involves prolonged cooking of the seeds by boiling and roasting with or without the shell. These processing methods usually improve the organoleptic properties of the nuts, reduce their antinutrients content, and prolong their shelf life (Ayankunbi, Keshinro, & Egele, 1991).

Thermal or heat processing is one of the most important methods developed by humans. During thermal processing, eventhough antinutritional components are reduced or eliminated; heat has a detrimental effect on the nutritional and functional properties of foods (Kanu et al., 2015). Then, the thermal treatment of walnuts can lead to chemical changes that can affect its nutritional value and the quality of its lipids. During boiling and roasting of the seeds, high temperatures can facilitate lipid oxidation and nonenzymatic browning reactions, which can reduce the nutritional value of foods, causing the loss of essential fatty acids, essential aminoacids and carbohydrates. The amount of vitamins can also be reduced as well as the proteins digestibility (Cuvelier & Maillard, 2012). Additionally, these chemical alteration reactions may generate toxic compounds in edible seeds and the derived products, which can be harmful for the consumers (Djikeng et al., 2017).

*Tetracarpidium conophorum* is cultivated in littoral and western Cameroon, where it is, respectively, known as “*kaso”* or “*ngak*.*”* The seeds are boiled and roasted for commercialization, consumption, and biscuit‐like snack food production (Babalola, 2011). Many studies have reported the effect of processing temperature on the nutritional, antinutritional values, and antioxidant properties, and African walnut seeds in some countries. These include: effect of boiling and traditional roasting on the nutritional, antinutritional, and antioxidant properties of African walnuts seeds (Arinola & Adesina, 2014); impact of processing on the nutrient content, vitamin, and mineral composition of African walnuts (Okonkwo & Ozoude, 2014); effect of cooking on phenolic content and antioxidant properties of African walnuts seeds Ademiluyi, Oboh, Aragbaiye, Oyeleye, and Ogunsuyi (2015). Though considerable attention had been given to the study of African walnut seeds, there is, however, very limited reports on the effects of processing on the walnut seeds grown in Cameroon, and especially on the quality of their lipids. Therefore, the objective of this study was to evaluate the effect of boiling and different roasting methods on lipid quality and proximate composition of walnut seeds.

## 2 MATERIAL AND METHODS

### 2.1 Material

**Figure 1:**
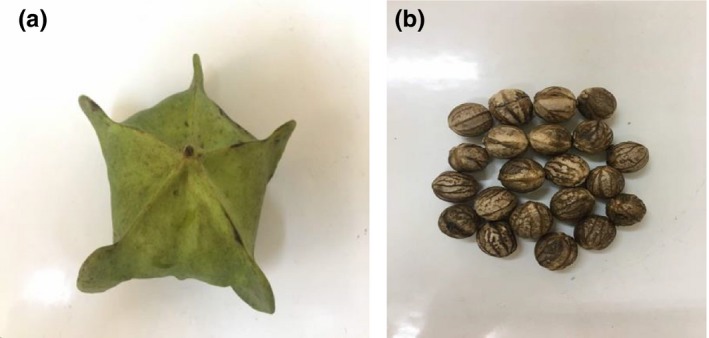
(a) Fresh walnut fruit; (b) Dried and unshelled walnut seeds

The fresh walnut seeds (*Tetracarpidium cornophorum*) (Figure 1) were harvested in the forest at Kumba, South‐West region, Cameroon in July 2017. All the chemicals and reagents used were of analytical reagent grade.

### 2.2 Methods

#### 2.2.1 Sample preparation and processing

The walnut seeds (all with the shells) were first cleaned and divided into 02 groups. The first group (G1) was processed fresh, and the second (G2) was dried to constant weight in an electric air‐dried oven at 50°C for 7 days before processing.

Group 1 samples (600 g) were divided into 03 identical subgroups of 200 g each, coded *FBWN* 60 min, *TRFWN* 30 min, and *FWN*. *FBWN* 60 min was boiled in 2 L of water at about 100°C for a period of 60 min. *TRFWN* 30 min was traditionally roasted in a cooking pot at 200–220°C by continous stirring for 30 min and *FWN* served as fresh control.

Group 2 samples (800 g) were divided into 04 identical subgroups of 200 g each, coded *DBWN* 60 min*, DTRWN* 60 min*, DORWN* 60 min, *and DWN. DBWN* 60 min was boiled in 2 L of water at about 100°C for a peroid of 60 min, *DTRWN* 60 min was traditionally roasted by continous stirring in a cooking pot at 200–220°C for 60 min, *DORWN* 60 min was also roasted for 60 min, but in the oven at 180°C, and *DWN* served as dry control.

### 2.3 Oil extraction

#### 2.3.1 Maceration method

This method was used for oil extraction from dried samples (*DTRWN* 60 min, *DORWN* 60 min, and *DWN*) as described by Womeni et al. (2016). The nuts were separately grinded to pass 1 mm sieve. A quantity of 80 g of each power was separately macerated in 400 ml of hexane at room temperature for 24 hr with constant shaking. After that, the mixture was filtered using the whatman paper N1, and the filtrate was concentrated on a rotatory evaporator at 40°C. The extracted oils were stored in the refrigerator at 4°C for further analysis.

#### 2.3.2 Bligh and dyer method

Oils were extracted from samples containing water (*FBWN* 60 min*, TRFWN 30 min, FWN*,* and DBWN* 60 min) using the method described by Bligh and Dyer (1959). About 80 g of nuts were introduced in a grinding machine (Moulinex) to which 100 ml of chloroform and 200 ml of methanol were subsequently added. The mixture was grinded for 3 min; follow by the addition of 100 ml of chloroform and 100 ml of water. The mixture was again grinded for 1 min, and filtered. The final extraction was ensured by the addition of chloroform, this in order to respect the following proportion: 2:2:1.8 for chloroform, methanol, and water, respectively. After separating the different phases in a funnel, the organic phase was collected and dried using sodium anhydrous. The organic solvent was then eliminated by evaporation on a rotatory evaporator at 45°C under reduced pressure. The extracted oils were stored in the refrigerator at 4°C for further analysis.

#### 2.3.3 Effect of processing on the quality of walnut oil

The determination of the peroxide value of walnut oil samples was made following the spectrophotometrical IDF standard method, 74A: 1991 (IDF, 1991). Its iodine and acid values were determined according to the procedure of AOCS Official Method CD 1‐25 and CD 3d‐63 respectively (AOCS, 2003). Finally, its thiobarbituric acid value was evaluated as described by Draper and Hadley (1990).

#### 2.3.4 Effect of different processing methods on the proximate composition of walnut

Moisture, fat, ash, and protein content of all the samples were determined using standard analytical methods described by AOAC procedures (AOAC, 1990). Moisture content was determined by drying walnut seeds in oven at 103°C until a constant weight was achieved according to the AOAC procedures 925.40 (AOAC, 1990). Ash content was determined by incineration of walnut seeds at 550°C according to the AOAC procedures 942.05 (AOAC, 1990). Nitrogen (N) content was determined using micro‐Kjeldahl method, according to AOAC procedures 984.13 (AOAC, 1990), the protein content was calculated as N × 6.25. Lipid content was determined using Soxhlet apparatus with hexane, following AOAC 963.15 methodology (AOAC, 1990). The total percentage carbohydrate content was determined by the difference method as reported by Onyeike et al. (2015). This method involved adding the total values of crude protein, crude fat, moisture and ash constituents of the sample and subtracting it from 100. All samples were analyzed in triplicate.

#### 2.3.5 Effect of processing on the mineral composition of walnut

For the determination of minerals, walnut seeds were ashed at 550°C and the ash boiled with 10 ml of 20% HCl in a beaker and then filtered into a 100 ml standard flask to determine the mineral content. Calcium (Ca), magnesium (Mg), sodium (Na), potassium (K), and iron (Fe) were determined by atomic absorption spectrometer (Varian 220FS Spectra AA, Les Ulis, France). Phosphorus (P) was determined colorimetrically using the vanado molybdate, according to AOAC procedure 965.17 (AOAC, 1999). Mineral contents of the samples were determined from calibration curves of standards minerals. All samples were analyzed in triplicate.

### 2.4 Statistical analysis

Results obtained in this study were subjected to one‐way analysis of variance (ANOVA) with Student–Newman–Keuls tests using Graphpad‐InStat version 3.05, to evaluate the statistical significance of the data. A probability value at *p* < .05 was considered statistically significant.

## 3 RESULTS AND DISCUSSION

### 3.1 Effect of processing on the quality of Walnut oil

#### 3.1.1 Peroxide value

Peroxide value (PV) is commonly used to determine the magnitude of primary oxidation products (mainly hydroperoxides) in oils (Shahidi & Wanasundara, 2008). The changes in PV of walnut oil samples are presented in Table 1. The peroxide values of all the processed samples (*DWN, DBWN* 60 min*, FBWN* 60 min*, DTRWN* 60 min*, DORWN* 60 min, *and TRFWN* 30 min) have significantly increased (*p* < .05) compared to that of the control (FWN). The highest PV was registered in *DBWN* 60 min*,* and was 19.10 meq O_2_/Kg, which was significantly higher than 10 meq O_2_/Kg, which is the recommended peroxide value of oils and fats (FAO/WHO, 2009). However, the PV of control and the other processed samples were lower than the recommended value, meaning that these treatments were better in preserving walnut oil than *DBWN* 60 min. No significant difference (*p* > .05) was observed between the PV of *FBWN* 60 min*, DTRWN* 60 min*, DORWN* 60 min, and *TRFWN* 30 min. The increase in peroxide value in the processed samples compared to the control *(FWN)* can be attributed to the accumulation of hydroperoxides as a result of free radicals attacking the unsaturated fatty acids of oil (Nyam, Wong, Long, & Tan, 2013; Womeni et al., 2016). These results are in accordance with those of Tenyang et al. (2017) who demonstrated that, the peroxide values of sesame oil increases with the thermal treatment of sesame seeds.

**Table 1:** Changes in peroxide, TBA, Iodine, and Acid values of walnut seed oil samples during processing

Samples	Peroxide value (meq O_2_/Kg)	TBA value (ppm)	Iodine value (g I_2_/100 g)	Acid value (mg KOH/g)
FWN	4.77 ± 0.00^d^	2.03 ± 0.54^e^	121.81 ± 0.02^a^, ^b^	1.17 ± 0.00^b^
DWN	5.42 ± 0.59^c^	2.17 ± 0.31^e^	120.49 ± 0.22^d^	0.92 ± 0.03^c^, ^d^, ^e^
DBWN 60 min	19.10 ± 0.10^a^	10.16 ± 0.26^a^	118.97 ± 0.06^f^	2.19 ± 0.10^a^
FBWN 60 min	9.02 ± 0.61^b^	6.17 ± 0.11^c^	120.04 ± 0.00^e^	1.11 ± 0.02^b^
DTRWN 60 min	8.00 ± 1.65^b^	7.49 ± 0.52^b^	120.78 ± 0.05^d^	1.04 ± 0.04^b^^,^ d
DORWN 60 min	7.44 ± 1.86^b^	5.21 ± 1.08^c^	121.68 ± 0.10^b^^,^ c	1.07 ± 0.25^b^^,^ c
TRFWN 30 min	7.48 ± 1.22^b^	3.42 ± 0.37^d^	121.52 ± 0.01^c^	1.12 ± 0.06^b^

Data are presented as mean ± SD (n = 3).

FWN , Fresh walnuts; DWN , Dried walnuts; DBWN 60 min , Dried and boiled walnuts (60 min); FBWN 60 min , Boiled fresh walnuts (60 min); DTRWN 60 min , Dried and traditionally roasted walnuts (60 min); DORWN 60 min , Dried and oven roasted walnuts (60 min); TRFWN 30 min , traditionally roasted fresh nuts (30 min).

^a^‐^f^Means within each column for parameter with different superscripts are significantly (*p* < 0,05) different.

#### 3.1.2 TBA value

Thiobarbituric acid value (TBA) value measures secondary oxidation production mainly malonaldehyde, which may contribute off‐flavor to oxidized oil (Djikeng et al., 2017). The effect of different processing methods on the TBA value of walnut oil is shown in Table 1. No significant difference (*p* > .05) was registered between the TBA values of *FWN* and *DWN*. However, a significant increase (*p* < .05) in this parameter was observed in the boiled and roasted samples compared to *FWN* and *DWN. DBWN* 60 min who previously exhibited the highest peroxide value, also presented the highest TBA value. This indicates that, the rate of primary and secondary oxidation in this sample was significantly higher (*p* < .05). This is the confirmation that, drying + boiling 60 min (*DBWN* 60 min) has significantly alters the quality of walnut oil compared to other treatments. The increase in TBA value registered in the cooked samples is the consequence of the formation of malonaldehyde, which is a secondary oxidation product obtained from the decomposition of hydroperoxides (Womeni et al., 2016). The fact that the cooking processes can increase the rate of production of secondary oxidation products in edible seeds has already been demonstrated. Tenyang et al. (2017) showed that the *p*‐anisidine value of sesame oil significantly increase with roasting and boiling temperatures and times.

#### 3.1.3 Iodine value

The iodine value (IV) generally determines the degree of unsaturation of edible oils and fats. A decrease in this parameter is generally attributed to the destruction of the double bonds of polyunsaturated fatty acids by free radicals (Tynek, Hazuka, Pawlowicz, & Dudek, 2001). The changes in IV of walnut oil samples during processing are presented in Table 1. Generally, the treatments have significantly decreased (*p* < .05) the iodine value of walnut oil compared to the control (*FWN*). The most affected sample was *DBWN* 60 min, because, its iodine value decreased from 121.81 (iodine value of the control) to 118.97 g I_2_/100 g. The significant decrease in iodine value of all the processed samples can be attributed as previously mentioned to the destruction of the double bonds of their unsaturated fatty acids by the free radicals formed during processing. The fact that the decrease was mostly pronounced in *DBWN* 60 min indicates a serious alteration of the unsaturations of its fatty acids compared to the other processed samples. It is important to note that, the iodine values obtained in almost all the samples were not far from the range 120 to 155 g I_2_/100 g which is the recommended range of iodine value of walnut oil (Soap Dish, 2002). As previously observed with the TBA and PV values, *DBWN* 60 min was the most affected sample. This means that, drying + boiling is not suitable for the preservation of the quality of walnut oil. These results are not in accordance with those of Tenyang et al. (2017) who demonstrated that, the iodine value sesame oil significantly decreases when the seeds are roasted at 120°C for 30 min.

#### 3.1.4 Acid value

The increase in acid value (AV) of oils might be an important measure of rancidity of foods. Free fatty acids are formed due to hydrolysis of triglycerides and may get promoted by reaction of oil with moisture (Frega, Mozzon, & Lercker, 1999). The changes in acidity of walnut oil during processing are shown in Table 1. No significant difference (*p* > .05) was registered between the control *(FWN)* and the following processed samples *(FBWN* 60 min*, DTRWN* 60 min*, DORWN* 60 min, and *TRFWN* 30 min). However, there was a significant increase (*p* < .05) in acid value of *DBWN* 60 min; and a significant decrease in AV of *DWN* compared to the control *(FWN)* and the other processed samples. The significant increase in acid value registered in *DBWN* 60 min might be attributed to the rapid hydrolysis of its triglycerides, leading to the accumulation of free fatty acids. It has been proven that, free fatty acids easily undergo oxidation than esterified ones. This can explain the fact that the lipids of this same sample were the most altered as previously observed with the PV, TBA, and IV. Also, its AV was lower than 4 mg KOH/g which is the recommended acid value of crude oils (FAO, WHO, 2009). The decrease in AV observed in *DWN* compared to *FWN* can be related to the rapid transformation of the free fatty acids present into hydroperoxides. Moreover, drying + boiling (60 min) significantly increase the acidity of walnut oil. This result is in accordance with those of Tenyang et al. (2017) who showed that the acid value of sesame oil significantly increases during boiling and roasting at different temperature and time.

### 3.2 Effect of processing on the proximate composition of walnut seeds

The effect of boiling and roasting on the proximate composition of walnut seeds is presented in Table 2. Results showed that, the moisture content of control *(DWN)* and processed walnut samples falls between 0.47 and 4.81%. *DTRWN* 60 min and *DORWN* 60 min have presented significantly lower (*p* < .05) amounts of moisture compared to the control (DWN) and the other processed samples. However, the moisture content of those other processed samples (*DBWN* 60 min*, FBWN* 60 min, *and TRFWN* 30 min was significantly higher (*p* < .05) than that of *DWN*. It has been demonstrated that, low moisture content of food samples increase their shelf‐lives by reducing the microbial and enzymatic activities (Oyenga, 2013). Concerning the changes in ash content, it was ranged between 7.30 and 9.03%. No significant difference (*p* > .05) was observed between the amount of ash of *DWN* (control) and those of *DBWN* 60 min*, FBWN* 60 min*, DORWN* 60 min, and *TRFWN* 60 min. However, a significantly higher (*p* < .05) ash content (9.03%) was registered with *DTRWN* 60 min, but that amount of ash was similar (*p* > .05) to that of *DBWN 60 min* (7.65%). This result suggests that drying + traditional roasting increases the amount of ash in walnut. The concentration of ash detected in this study is higher than those reported by Arinola and Adesina (2014). These authors reported that the amount of ash in raw, boiled and roasted walnut was ranged between 2 and 4%, and was decreasing with the treatments. However, our findings were not far from those of Onyeike, Anyalogbu, and Monanu (2015) who demonstrated that ash content of cooked and raw walnut seeds of 5.45%–6.05%. They also find no significant difference between the amount of ash in cooked and raw walnut.

**Table 2:** Changes in proximate composition of walnut seeds during processing

Samples	Moisture%	Ash%	Lipid%	Protein%	Carbohydrate%
DWN	1.79 ± 0.08^c^	7.35 ± 0.35^b^	52.75 ± 0.20^c^	24.18 ± 0.83^a^	13.09 ± 0.06^b^
DBWN 60 min	3.78 ± 0.00^b^	7.65 ± 0.63^b^, ^a^	52.98 ± 0.00^c^	21.96 ± 0.06^b^	12.67 ± 0.21^c^
FBWN 60 min	3.67 ± 1.69^b^	7.70 ± 0.28^b^	56.89 ± 0.08^b^	23.26 ± 0.08^b^	9.21 ± 0.09^c^
DTRWN 60 min	0.49 ± 0.00^d^	9.03 ± 0.36^a^	56.14 ± 0.12^b^	20.16 ± 0.12^c^	14.18 ± 0.11^a^
DORWN 60 min	0.47 ± 0.00^d^	7.95 ± 0.35^b^	56.27 ± 0.55^b^	22.69 ± 0.01^b^	12.23 ± 0.05^d^
TRFWN 30 min	4.81 ± 0.00^a^	7.30 ± 0.46^b^	54.29 ± 0.03^b^	23.16 ± 0.12^a^	11.44 ± 0.03^e^

Data are presented as mean ± SD (n = 3).

DWN, Dried walnuts; DBWN 60 min, Dried and boiled walnuts (60 min); FBWN 60 min, Boiled fresh walnuts (60 min); DTRWN 60 min, Dried and traditionally roasted walnuts (60 min); DORWN 60 min, Dried and oven roasted walnuts (60 min); TRFWN 30 min, traditionally roasted fresh nuts (30 min).

^a^‐^f^Means within each column with different superscripts are significantly (*p* < .05) different.

Results also showed that walnut seeds are rich in macronutrients: 52.75%–56.89% of lipids, 21.96%–24.18% of proteins and 9.21%–14.18% of carbohydrates (digestibles and undigestibles). This composition is closed to that previously reported by Arinola and Adesina (2014) with the same nuts. These authors showed that the total lipids, proteins and carbohydrates content of cooked and raw walnut varied between 54.14 and 62.65%, 22.47 and 29.14%, and 11.41 and 13.4%, respectively. It is also observed in Table 2 that the amounts of lipid of *FBWN* 60 min*, DTRWN* 60 min*, DORWN* 60 min, and *TRFWN* 30 min were significantly higher (*p* < .05) compared to that of *DWN* (control) and *DBWN* 60 min. This might be due to the loss of water, which facilitate lipids extraction. However, no significant difference (*p* > .05) was registered between the lipid content of *DWN* and *DBWN* 60 min. Similar trend has been obtained by Onyeike et al. (2015) when evaluating the effect of heat processing on the proximate composition and energy value of African walnut seeds. Talking about the total protein content, a significant decrease (*p* < .05) in its concentration was registered with *DBWN* 60 min*, DTRWN* 60 min, and *DORWN* 60 min compared to the control (DWN). This reduction can be attributed to the Maillard reaction, as proteins are substrates of nonenzymatic browning (Tenyang et al., 2017). Similar result has been reported by Arinola and Adesina (2014), who showed that the amount of protein of walnut seed was decreasing during boiling and roasting. However, no difference was registered between the amount of protein of *FBWN* 60 min*, TRFWN* 30 min, and *DWN* (Control). The total carbohydrate content of processed and raw walnut sample also presented in Table 2 showed that, the amount of sugar varies significantly (*p* < .05) with the processing method. The amount of carbohydrate of *DBWN* 60 min*, FBWN* 60 min, *DORWN* 60 min, and *TRFWN* 30 min has significantly (*p* < .05) decreased during the treatments compared to the control *(DWN)*. As previously mentioned, this can be related to the Maillard reaction, as carbohydrates (reducing sugars) are also substrates of nonenzymatic browning (Tenyang et al., 2017). These results are in accordance with those of Onyeike et al. (2015) who demonstrated that the total carbohydrate content of walnut seeds was decreasing during processing.

### 3.3 Effect of processing on the mineral content of walnut seeds

The changes in mineral composition of walnut seeds during processing are presented in Table 3. It can be observed that the nuts contain important amount of macrominerals, among which calcium, magnesium, and potassium are the most represented. Sodium and phosphorus were also present, but at low concentration compared to the previous minerals. The importance of these mineral elements in human health has already been proven. They are implicated in several body functions such as enzymatic reactions, energy production, transmission of nerve impulses, and multiple biological reactions (Steinberg, Bearden, & Keen, 2003). The data of calcium shows that its amount has significantly decreased (*p* < .05) during cooking compared to the *control (DWN)*. The lowest amounts of calcium were found in *DBWN* 60 min and *FBWN* 60 min*,* and were 569.00 and 671.50 mg/100 g, respectively. Globally, the concentration of calcium obtained in this study is higher than 433.5 mg/100 g as reported by Ayoola, Onawumi, and Faboya (2011). This mineral, together with the phosphorus are very essential for bone metabolism (Nwaoguikpe, Ujowundu, & Wesley, 2012).

**Table 3:** Changes in mineral composition of walnut during processing

Samples	Fe (mg/100 g)	Ca (mg/100 g)	Mg (mg/100 g)	K (mg/100 g)	Na (mg/100 g)	*p* (mg/100 g)
DWN	18.54 ± 0.74^d^	1969.00 ± 12.72^a^	1119.35 ± 3.04^c^	951.72 ± 8.16^a^	76.75 ± 2.47^a^	21.34 ± 0.33^a^, ^b^
DBWN 60 min	24.81 ± 0.40^b^	568.00 ± 11.31^e^	172.85 ± 3.88^f^	543.02 ± 3.50^c^	54.00 ± 5.65^b^	18.92 ± 0.04^a^, ^c^
FBWN 60 min	20.84 ± 0.32^c^	671.50 ± 4.94^d^	203.34 ± 2.34^e^	744.52 ± 1.80^b^	53.90 ± 5.51^b^	22.38 ± 0.22^a^
DTRWN 60 min	16.09 ± 0.96^e^	1566.25 ± 8.83^b^	1539.05 ± 6.29^b^	744.87 ± 2.29^b^	53.50 ± 4.94^b^	16,78 ± 0.16^c^
DORWN 60 min	43.32 ± 0.80^a^	1427.25 ± 10.25^c^	1691.35 ± 20.71^a^	747.02 ± 5.33^b^	53.00 ± 4.24^b^	16.87 ± 0.29^c^
TRFWN 30 min	14.64 ± 0.36^e^	1411.50 ± 16.26^c^	416.05 ± 4.17^d^	544.02 ± 4.91^c^	54.25 ± 6.01^b^	16.86 ± 0.27^c^

Data are presented as mean ± SD (*n* = 3).

DWN, Dried walnuts; DBWN 60 min, Dried and boiled walnuts (60 min); FBWN 60 min, Boiled fresh walnuts (60 min); DTRWN 60 min, Dried and traditionally roasted walnuts (60 min); DORWN 60 min, Dried and oven roasted walnuts (60 min); TRFWN 30 min, traditionally roasted fresh nuts (30 min).

^a^‐^f^Means within each column with different superscripts are significantly (*p* < .05) different.

Concerning the magnesium content, its concentration significantly decreased (*p* < .05) in *DBWN* 60* *min*, FBWN* 60* *min, and *TRFWN* 30* *min (172.75, 203.34, and 416.05 mg/100 g, respectively) compared to *DWN* (1119.35 mg/100 g), while those of *DTRWN* 60 min and *DORWN* 60 min were significantly higher (1539.05 and 1691.35 mg/100 g, respectively). This indicates that, oven and traditional roasting for 10 min is suitable for the elevation of the amount of magnesium in walnut seeds. The decrease in magnesium content registered in *DBWN* 60 min*, FBWN* 60 min, and *TRFWN* 30 min can be attributed to the loss of this mineral in water during processing through the diffusion process. However, its elevation in *DTRWN* 60 min and *DORWN* 60 min can be due to the fact that, the antinutritional present in the nuts and which were complexed to the mineral were significantly destroyed by heat, leading to the increase in its concentration (Makinde & Akinoso, 2013). The amount of magnesium obtained in this study was higher than 171.12 mg/100 g as previously reported by Ayoola et al. (2011) in the same nuts.

From Table 3, we can also see that the amount of potassium and sodium has significantly decreased (*p* < .05) during cooking compared to the control (DWN). However, their concentrations were significantly higher than 625 mg/100 g and 26 mg/100 g as reported by Ayoola et al. (2011) and Nwaoguikpe et al. (2012), respectively. The presence of these minerals in African walnut is also beneficial, due to their direct relationship with hypertension in humans. This may be the reason why the plant is used to prevent and control high blood pressure (James, 2000). The phosphorus content of walnut was also reducing in almost all the cooked samples. Its concentration was ranged between 16.86 and 22.38 mg/100 g, which is lower than 35 mg/100 g as previously reported by (Nwaoguikpe et al., 2012).

Concerning the single micronutrient analyzed, (iron) its concentration has significantly increased (*p* < .05) in DBWN 60* min,* FBWN 60 min, and DORWN 60 min compared to the control. However the amount of iron in DTRWN 60 min and TRFWN 30 min were significantly lower than those previously mentioned. This indicates that traditional roasting significantly decreases the amount of iron of walnut. The concentrations of iron obtained in this study vary between 14.64 and 43.32 mg/100 g, which is higher than 11.0 mg/100 g as previously reported by Oyoola et al. (2011). The amount of iron provided by walnut can helps in the prevention of iron‐nutritional anemia.

## 4 CONCLUSION

The objective of this study was to evaluate the effect of boiling and roasting on lipid quality and nutritional value of walnut seeds. Results show that the quality of walnut oil was significantly affected by boiling and roasting. However *DBWN* 60 min and *FBWN* 60 min were the most altered samples. These treatments are not suitable for the preservation of walnut oil quality. The proximate composition of walnut seeds was also significantly affected by the treatments. Almost all the cooking methods have significantly increased the lipid content of the nuts, while their protein and carbohydrate concentrations decreased. Traditional roasting for 60 min increases the ash content of walnut seeds. The minerals of the nuts were also significantly modified during cooking. Both boiling and roasting have significantly decreased the K, Ca, Na, and P content of walnut. Traditional and oven roasting for 60 min considerably increase the amount of magnesium, while the other treatments decrease its concentration. All processing technique including boiling leads to a significant loss of calcium and magnesium. Traditional roasting reduces the concentration of iron of walnut seeds.

## CONFLICT OF INTEREST

None declared.
